# Metagenomics by next-generation sequencing (mNGS) in the etiological characterization of neonatal and pediatric sepsis: A systematic review

**DOI:** 10.3389/fped.2023.1011723

**Published:** 2023-03-30

**Authors:** Sergio Agudelo-Pérez, Jaime Fernández-Sarmiento, Diana Rivera León, Ronald Guillermo Peláez

**Affiliations:** ^1^ Department of Pediatrics, Faculty of Medicine, Universidad de La Sabana, Chia, Colombia; ^2^ Departament of Pediatrics and Critical Care, Fundación Cardioinfantil, Bogotá, Colombia; ^3^ Life Sciences and Health Research Group, Graduates School, CES University, Medellin, Colombia

**Keywords:** next generation sequencing - NGS, metagenomics, high-Throughput nucleotide sequencing, sepsis, newborn, children

## Abstract

**Introduction:**

Pediatric and neonatal sepsis is one of the main causes of mortality and morbidity in these age groups. Accurate and early etiological identification is essential for guiding antibiotic treatment, improving survival, and reducing complications and sequelae. Currently, the identification is based on culture-dependent methods, which has many limitations for its use in clinical practice, and obtaining its results is delayed. Next-generation sequencing enables rapid, accurate, and unbiased identification of multiple microorganisms in biological samples at the same time. The objective of this study was to characterize the etiology of neonatal and pediatric sepsis by metagenomic techniques.

**Methods:**

A systematic review of the literature was carried out using the PRISMA-2020 guide. Observational, descriptive, and case report studies on pediatric patients were included, with a diagnostic evaluation by clinical criteria of sepsis based on the systemic inflammatory response, in sterile and non-sterile biofluid samples. The risk of bias assessment of the observational studies was carried out with the STROBE-metagenomics instrument and the CARE checklist for case reports.

**Results and Discussion:**

Five studies with a total of 462 patients were included. Due to the data obtained from the studies, it was not possible to perform a quantitative synthesis (meta-analysis). Based on the data from the included studies, the result identified that mNGS improves the etiological identification in neonatal and pediatric sepsis, especially in the context of negative cultures and in the identification of unusual microorganisms (bacteria that are difficult to grow in culture, viruses, fungi, and parasites). The number of investigations is currently limited, and the studies are at high risk of bias. Further research using this technology would have the potential to improve the rational use of antibiotics.

## Introduction

Sepsis is one of the main causes of morbidity and mortality in the world (1). For example, in the group of premature infants, it is the main cause of mortality and morbidity (2), causing approximately 3 million deaths per year (3). In pediatric patients, it has become a public health problem with mortality ranging between 5%–20%, depending on the context of care and comorbidities (1). The burden of disease and sequelae occurs mainly in countries with low and middle incomes (4, 5). Additionally, the disease poses a challenge to the clinician in the neonatal and pediatric intensive care unit in terms of approach and initial diagnosis and in the timely establishment of management that allows mortality and complications to be reduced.

On the other hand, there are current problems in the initial approach to patients. For example, the gold standard for microorganism identification and diagnosis is sterile site culture (blood, urine, and/or cerebrospinal fluid) (6, 7), and the etiology and epidemiology have traditionally been established based on this pattern of reference, thus guiding the initial empirical treatment (8, 9). However, this diagnostic method exhibits several limitations. First, for diagnosis, it has low sensitivity and specificity, with a high rate of false positives and negatives (10), which complicates the interpretation of the result in clinical practice. Additionally, the identification of the microorganism causing sepsis takes days, delaying the orientation of targeted treatment (11). In this sense, although the initial empiric antibiotic orientation is essential, the etiological identification of the microorganism is also essential for an adjustment of the therapy and/or timely discontinuation of the antibiotic treatment to reduce complications and improve the course of the disease (12–14). Therefore, given the need for rapid diagnosis, early initiation of targeted antibiotic therapy, and prompt clinical stabilization of associated medical conditions as a fundamental measure to improve survival, these limitations hinder initial patient management, increasing the risk of death and complications (15, 16).

Likewise, the culture methods used in the clinical laboratory allow the growth and identification of a reduced spectrum of microorganisms, leaving out other non-cultivable or difficult-to-grow ones that could be of interest in the etiology of neonatal and pediatric sepsis (17, 18). In addition, culture-based characterization of the etiology has identified sepsis in these age groups as monomicrobial (19). In contrast, some studies with culture-independent technologies, such as molecular techniques, have also shown the probability of a polymicrobial infection (17, 19).

So, currently, the recommendations for initial empirical antibiotic management are based on the etiological characterization identified by culture-dependent methods (20–22), and the targeted orientation of antimicrobial treatment is not achieved until the causal agent is identified in the culture. This situation can lead to non-specific treatment in patients with sepsis, unnecessarily exposing patients to broad-spectrum empirical antibiotics (18, 23), increasing the risk of medical complications in the different age groups affected (example, necrotizing enterocolitis, invasive fungal infection), and the development of microbial resistance to antibiotics, alterations in the development of the intestinal microbiota, and death (13, 14).

On the other hand, there are currently new culture-independent technologies, such as molecular methods based on PCR (polymerase chain reaction), that make it possible to amplify and detect genes or gene regions exclusive to one or several sepsis-causing microorganisms. In addition, new generation sequencing (NGS) technologies used to amplify gene regions such as 16S (bacteria), 18S (fungus), ITS (Internal Transcribed Spacer), or genes encoding viral proteins have allowed the simultaneous identification of a wide spectrum of sepsis-causing microorganisms in a single sample. Next-generation sequencing (NGS) describes several methods to sequence RNA and DNA at a faster pace and cheaper cost. Similarly, it is a term to describe very high throughput sequencing methods that allow millions of fragments to be made in parallel during a single run (24). For this, NGS platforms can use different technologies to perform sequencing, then by bioinformatics analyses, these fragments piece together by mapping the individual reads to the reference genome (25). The clinical uses of NGS include clinical genetics, oncology, and microbiology among others. On other hand, metagenomics refers to the use of NGS to simultaneously identify genomic material from all organisms present in a sample and could detect all potential pathogens (bacteria, viruses, fungi, and parasites) (26, 27). Applications include pathogen detection and discovery, species characterization, antimicrobial resistance detection, virulence profiling, and study of the microbiome and microecological factors affecting health (26). Additionally, transcriptome sequencing methods (RNAseq) have allowed the identification of genes with an increase or decrease in their transcription level related to the immune response that can serve as biomarkers of inflammatory response in sepsis processes (11, 28). These techniques have the option of identifying non-culturable microorganisms and offer the probability of improving the ability to diagnose and track infectious diseases (29), allowing the clarification of the etiology of sepsis, which, in turn, can lead to the design of prevention strategies and optimal treatments for specific germs (19).

So, to face the challenge of rapidly identifying the sepsis-causing microorganism and guiding empiric and directed treatment quickly and appropriately to reduce complications and improve the course and outcome of the disease, the NGS technologies used to amplify gene regions could be an alternative for the initial approach to the patient. Therefore, this study was carried out to conduct a systematic review of published data regarding the etiological characterization of neonatal and pediatric sepsis by culture-independent methods based on metagenomic NGS (mNGS) techniques. Previously, we did not identify systematic reviews on this topic, so the review contributes to the characterization from the microorganisms involved in sepsis, allowing to identify the current state of research with mNGS in pediatrics and neonatology and could be the basis for designing research focused on improving antibiotic use guidelines and diagnosis in neonatal and intensive care units. In this scenario, the following research question was formulated: What is the etiological characterization of neonatal and pediatric sepsis identified by NGS (mNGS) metagenomic studies?

## Materials and methods

### Literature search strategy

A systematic review of the literature was conducted using the PRISMA-2020 (Preferred Reporting Items for Systematic Reviews and Meta-Analyses) guideline (30) for the identification, screening, and inclusion of articles. The search was carried out between September and November 2021 in the electronic databases PubMed, Embase, Scopus, and Web of Science. The search was not restricted by language or year, and observational studies (cases and controls, cohort, and cross-sectional studies), descriptive studies, and case reports or series were included.

The search terms used included synonyms or thesauri from the MeSH (Medical Subject Headings) web dictionaries: Sepsis, Metagenomics, and High-Throughput Nucleotide Sequencing. Additionally, generic terms such as RNAseq and Next Generation Sequencing were used. The following search strategy was used for PubMed and adapted to the other databases: *(sepsis) AND ((((RNAseq) OR (Metagenomics)) OR (High-Throughput Nucleotide Sequencing)) OR (Next Generation Sequencing))*.

### Study eligibility criteria

Inclusion criteria

1. Pediatric age population, considered from 0 days of life to 18 years of age. Specifically, for the newborn, the term newborn was considered (gestational age greater than or equal to 37 weeks of gestation) and up to 30 days of life: and preterm newborn (gestational age less than 37 weeks) and up to 30 days of life and/or 40 weeks of corrected age.
2. Having been included in the study for diagnostic evaluation by clinical criteria for sepsis based on the systemic inflammatory response. In the neonatal group, studies that evaluated the population at risk of perinatal infection for neonatal sepsis due to the presence of risks in the mother (e.g., premature rupture of ovular membranes, chorioamnionitis, preterm delivery) were also included.
3. The results of the identification of the microorganisms that cause sepsis through metagenomic techniques will be published.
4. Analysis of samples taken in sterile bio-fluids such as blood and/or serum, including umbilical cord, urine, and/or cerebrospinal fluid samples; or other non-sterile bio-fluids such as aspirate from respiratory secretions or feces.

Exclusion criteria

1. Literature reviews such as systematic, integrative, and/or narrative reviews; summary of conferences and correspondence to the editor.
2. Poster presentations, conferences, and/or abstracts only.
3. Animal studies.

The synthesis of the data and the characterization of the microorganisms by age group–newborn and pediatric–were carried out. For the neonatal group, we planned to categorize the data into term and preterm infants; and for early (first 72 h of life) and late (after 72 h of life) neonatal sepsis when information was available. Additionally, for the characterization of the information, it was planned to describe the data by type of sample (blood and/or serum, urine, and/or cerebrospinal fluid samples, etc.), group of microorganisms analysed and clinical characteristics of the patients.

### Screening and inclusion of studies

The initial search and selection of studies were carried out by the investigators independently (SA, JF, DR, RP). The identified studies were screened by title and abstract in the Rayyan® web tool (31), where duplicate records were also identified due to overlap between the databases consulted. Initial results were compared, and discrepancies were resolved by consensus by the researchers. To define their final entry into the systematic review, the articles identified as relevant to the systematic review by screening were retrieved in full text for in-depth reading by the three researchers independently. Discrepancies were resolved by consensus.

### Data extraction and synthesis

Information on the characteristics of the study was extracted in terms of (a) name of the journal, (b) author and year, (c) region of the study where it was carried out; and details of the population such as (d) term or preterm neonate, pediatric patient, (e) in the case of neonates with early or late sepsis, (f) clinical characteristics, (g) origin of the sample (bio- fluid), (h) microorganisms identified and whether the infection was polymicrobial or monomicrobial.

### Assessment of methodological quality

The assessment of the risk of bias in the observational studies was carried out with the STROBE-metagenomics instrument (STROBE extension statement to guide the report of metagenomic studies) (26) and the CARE checklist (32) for case reports. A quantitative meta-analysis was not performed for several reasons. Relative effect estimates could not be calculated for some observational studies and studies lacking control groups. Methodological heterogeneity, such as type of assessment, the timing of assessment relative to disease onset, and the wide range of factors associated with patients' sepsis did not allow quantification of results. Therefore, all studies were analysed qualitatively.

## Results

### Characteristics of included studies

**Figure 1:**
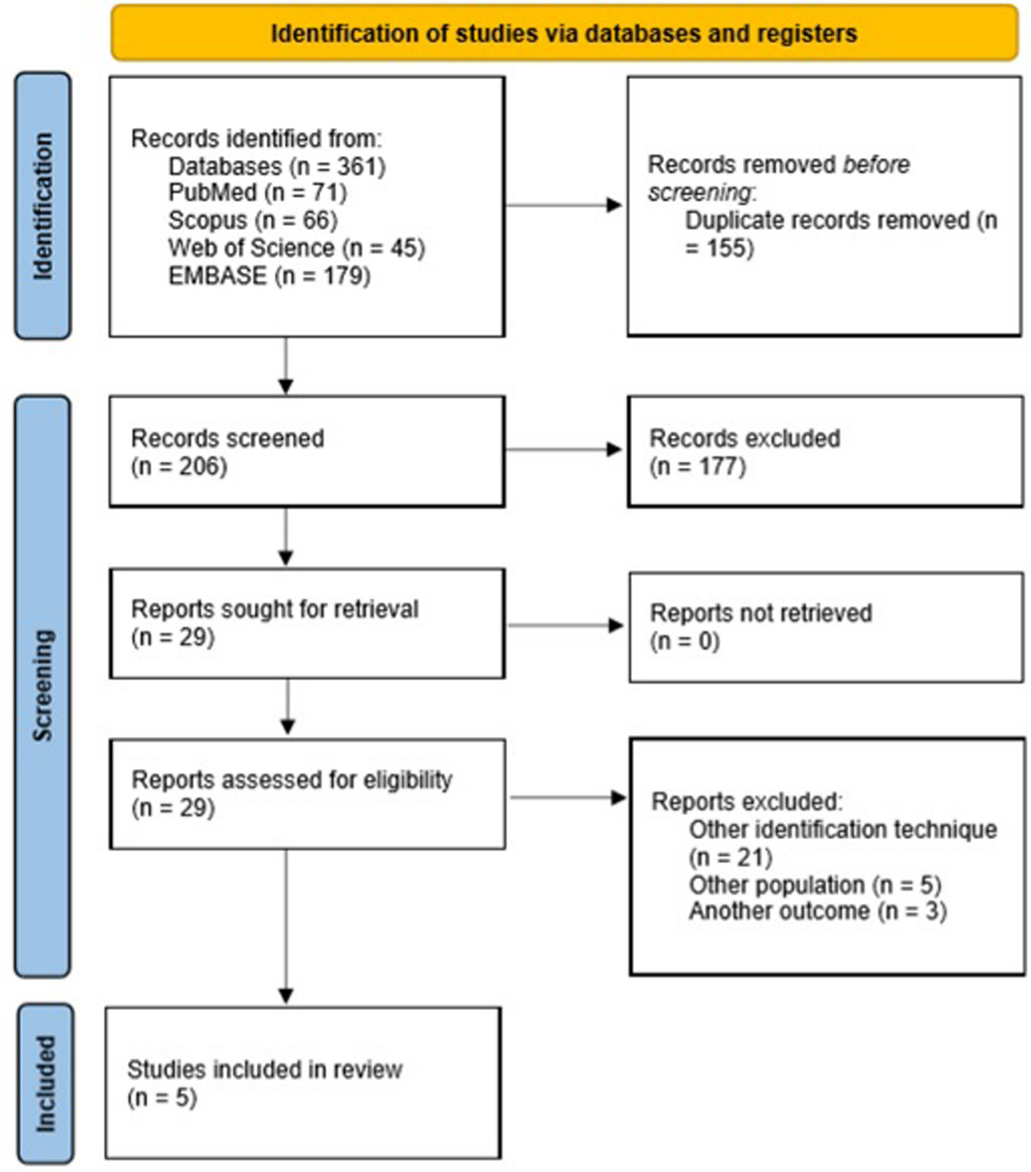
PRISMA Flow Diagram.

A total of *n* = 361 studies were identified. After eliminating duplicates (*n* = 155), *n* = 206 studies were evaluated in the initial screening, eliminating *n* = 177 (86%). One of the main causes of exclusion was due to other outcomes, including the use of NGS with other objectives, such as: RNA expression profiling in patients with sepsis, evaluation of gene regulation in sepsis models, molecular signalling pathways, genes and molecules involved in the inflammatory immune response, sequencing of the complete genome of bacteria and specific viruses isolated in the course of the infection, other types of infections other than a septic clinical (e.g., HIV, COVID-19, pneumonia, etc.), study of exomes in patients with sepsis, study of single nucleotide polymorphism (SNP) in sepsis, evaluation and study of virulence factors and resistance genes of specific microorganisms. Similarly, studies were excluded by adult population and studies in animals (animal models for human infections studies or primary studies of infections in animals). Concerning the exclusion of studies due to the technology used, it was mainly due to the use of culture-independent molecular tests other than mNGS, such as tests based on polymerase chain reaction (e.g., Multiplex, Septicyte, etc.). Additionally, other studies were excluded due to the use of NGS in other pathologies as oncology, haematology, and the study of inborn errors of immunity. Finally, after the in-depth reading, *n* = 5 studies (17%) were included [Figure 1]. Therefore, although most of the studies used molecular tools for different phases of the diagnosis and identification of microorganisms in sepsis, they were not the subject of this review.

One hundred percent of the articles included were published in English and are from the Asian continent (*n* = 3 from Japan, *n* = 1 from China, and *n* = 1 from Vietnam). Regarding the design of the studies, in three, an initial prospective design was proposed for the inclusion of the patients and the collection of the sample which were all from the pediatric population (33–35); however, in all the studies, the mNGS analysis of the collected samples was conducted retrospectively on the samples which were stored and frozen. Two studies were case reports which were from the neonate population (36, 37). The characteristics of the admitted studies are shown in Tables 1,2.

**Table 1:** Characteristics of the included studies.

Reference	Population	Microorganism (mNGS)	Study design	Methodology
Horiba et al. (36) 2021 Japan	Full Term Neonate *n* = 2	Bacterial Identification	Case Report	Sequencing platforms: the Illumina HiSeqX. Sequencing deep: no inform Bioinformatics analysis: - DNA sequencing libraries were prepared using a Nextera XT library Prep Kit (Illumina, San Diego, CA, USA). - Sequence data were processed using the metagenomic analysis pipeline PATHDET version 1.0
**Mariya et aln. (37) 2021 Japa**	Placenta of preterm pregnancy with chorioamnionitis and preterm newborns at risk of sepsis. *n* = 1: gestation of 14 weeks whit maternal sepsis. *n* = 2: term newborn without sepsis, as the negative control. *n* = 3 and *n* = 4 preterm at 26 and 24 weeks with PROM** and chorioamnionitis criteria	Bacterial Identification	Case Report	The V1–V2 hyper-variable region of the bacterial 16S rRNA gene was amplified from the extracted DNA by PCR. Sequencing platforms: Illumina MiSeq platform. Sequencing deep: no inform Bioinformatics analysis: - DNA sequencing libraries were prepared using a Illumina Nextera XT Kit (Illumina, San Diego, CA, USA). - Quality control for merged sequences was performed using PRINSEQ-lite-0.20.4 to truncate the primer-binding region and discard the lowquality sequences (Q score < 25, read length < 250 bp or > 400 bp). - Operational taxonomic units (OTUs) were created by pick_otus.py of QIIME 1.9.1 - To assign species level taxonomy, a homology search using BLAST was carried out on the representative sequence of each OTU based on the SILVA132 and STIRRUPS databases.
Horiba et al. (33) 2018 Japan	*n* = 35 Pediatric patients immunocompromised due to cancer treatment and central catheter. *n* = 12 with proven sepsis (positive culture) and *n* = 23 with suspected sepsis (negative culture).	Bacterial Identification	Prospective cohort *mNGS analysis was retrospective on frozen samples	Sequencing platforms: HiSeq2500 (Illumina). Sequencing deep: no inform Bioinformatics analysis: - DNA sequencing libraries were prepared using a Nextera XT library. - NGS data were processed using the cloud-computing pipeline for metagenomic identifying pathogens, MePIC v2.0 - Library quality was determined using an Agilent 2,200 TapeStation. - Reads were mapped against known nucleotide sequences in the database including microbial genomes using MEGABLAST programs. Calculated: - Bacterial reads per million reads of the sequence depth (BR). - Relative importance values of the dominant bacteria (P1) were calculated as percentages of the reads of the “dominant” bacteria. - Shannon's diversity index (H’) based on each taxonomic hierarchy was calculated from the bacterial reads. - ResFinder database was used to detect antimicrobial resistance genes.
**Anh et al. (34) 2019 Southeast Asia: Indonesia, Thailand, and Vietnam**	*n* = 386 *original study had *n* = 1,582 patients (adults 815—children 763) 54% (*n* = 402) had no etiology identified (negative culture) in the original study, of which *n* = 386 are included in the viral mNGS study	Viral Identification	Prospective cohort *mNGS analysis was retrospective on frozen samples originally taken for an etiology study in sepsis.	Sequencing platforms: Illumina MiSeq. Sequencing deep: no inform Bioinformatics analysis: - Library preparation using a Nextera XT sample preparation (Illumina). - The mNGS data were analyzed using an in-house viral metagenomic pipeline running on a 36-node Linux cluster to identify the presence of viral sequences in the tested specimens. - The resulting contigs and singlet reads were then aligned against a customized viral proteome database using a BLAST (Basic Local Alignment Search Tool)-based approach.
**Yan et al. (35) 2021 China**	Pediatric patients: > 28 days and < 18 years Admitted to the Pediatric Intensive Care Unit with a diagnosis of sepsis Total *n* = 34 *n* = 18/34 confirmed sepsis (*n* = 7 community-acquired pneumonia and *n* = 6 hospital-acquired pneumonia)	Identification of the complete microbiome: bacteria virus fungus parasites	Prospective cohort *mNGS analysis was retrospective on frozen samples	Sequencing platforms: BGISEQ-50 (MGI Technology, Shenzhen, China). Sequencing deep: single-end 50 bp Bioinformatics analysis: - Library: the MGIEasy Cell-free DNA Library Prep Kit. - 2,100 Bioanalyzer (Agilent, USA) was used for library quality control, and the qualified library fragment size was 200-300 bp. - Microbial Ecological Diversity: Species abundance, species count, Chao1 diversity index, and Shannon diversity index.

^a^ PROM: premature rupture of ovular membranes.

**Table 2:** Characterization of neonatal and pediatric sepsis

Reference	Sample	Clinical feature	Bacteria	Virus	Fungus	Parasite
Horiba et al. (36) 2021 Japan	CSF Blood	Newborn whit SIR. CSF cytochemical suggestive of meningitis. CSF and blood culture negative Empirical antibiotic formulation.	Streptococcus agalactiae in CSF Others: Other Streptococcus	N/A	N/A	N/A
Mariya et al. (37) 2021 Japan	Umbilical Blood Placenta	Preterm birth due to chorioamnionitis. The placenta is studied, and it is correlated with the development of neonatal sepsis (blood culture). A healthy-term pregnancy placenta control is included in the study.	*Streptococcus pyogenes* *Burkholderia* *Klebsiella* *Sphingomonas* *Enterobacter hormaechei* *Methylobacterium adhaesivum* *Corynebacterium tuberculostearicum* *Ralstonia pickettii* *Sphingopyxis macrogoltabida*	N/A	N/A	N/A
Horiba et al. (33) 2018 Japan	Blood	Sepsis criteria based on SIR. Sepsis criteria: pathogen detected in culture, fever > 38°C, C-Reactive protein > 1 mg/dl. Suspected sepsis: negative culture. Proven sepsis: positive culture.	There was identical identification of bacteria in culture and mNGS in 8 patients. However, mNGS detected other unidentified bacteria in culture. Indices for the etiological identification of sepsis: sequencing depth (BR) > 200 and relative importance value (P1) > 0.5 as recommended values for the etiological determination of the pathogen. Research of resistance genes: in 7 patients with sepsis and 2 with suspected sepsis, finding 62 resistance genes.	N/A	N/A	N/A
Anh et al. (34) 2019 Southeast Asia: Indonesia, Thailand, and Vietnam	Blood CSF Pharyngeal swab tracheal aspirate Stool	Patients in a general hospitalization service with a diagnosis of community-acquired sepsis. Definition criteria based on pediatric consensus for sepsis.	N/A	93% (*n* = 358/386) of patient's identification of viral sequences. *n* = 108 coinfection with > 2 viruses. The most frequent viruses identified are enteroviruses, hepatitis B virus, cytomegalovirus, rhinovirus, Ebstein-Barr virus, and rotavirus. The highest proportion of viral identification was in patients with central nervous system infection (37.5%), respiratory infection (23%), and patients with systemic infection (12.5%). Enteroviruses and respiratory viruses were the most frequently isolated in the pediatric population.	N/A	N/A
Yan et al. (35) 2021 China	Blood	Patients with SIR and international diagnostic criteria for sepsis. Analysis of serum inflammatory markers (LPS, CRP, and procalcitonin); lymphocyte subpopulation. The identification of fungal and parasitic sequences was significantly higher in patients with immunodeficient diseases. serum procalcitonin was positively correlated with bacterial abundance in the blood.	*Escherichia coli, Pseudomonas aeruginosa, Acinetobacter baumannii, and Klebsiella pneumoniae*. Significant correlation between the Chao1 index and the degree of bacteremia.	Pathogenic viruses detected: human mastadenovirus B, cytomegalovirus, and Ebstein-Barr virus. Significant positive correlation between inflammatory markers (IL-6 and CRP) and viral sequence identification.	Fungi (*n* = 12/34): Pneumocystis jiroveci, Saccharomyces cerevisiae, Aspergullus fischeri, Malazessia restricta. Correlation between leukopenia and Pneumocystis jiroveci.	Parasites: Plasmopara halstedii, Leishmania mexicana, Nannochloropsis gaditana, Eimeria acervulina. The richness of blood protozoa (Chao1) is higher in children with immunodeficiency than in those without immunodeficiency.

^a^ CSF: Cerebrospinal fluid; SIR: systemic inflammatory response.

The total number of patients included in the studies was *n* = 462, and nearly 99% were of pediatric age. In this sense, two studies are of infections in the perinatal period (*n* = 1 neonates and *n* = 1 preterm gestation complicated with chorioamnionitis) (36, 37). Most of the studies include a small number of patients, except for the study by Anh et al. (34), which analyses samples from 388 children. On the other hand, the analysed samples in the studies were blood (33–37) and cerebrospinal fluid (34, 36). The Anh study had the particularity of analysing, in addition to the blood samples and CFS, samples of the pharyngeal swab, tracheal aspirate and Stool (34). Regarding the objective in the detection of microorganisms by mNGS, three studies focused on the detection of bacteria (33, 36, 37), one on the detection of viruses (in this case they analysed samples from patients with sepsis criteria but with negative cultures) (34) and only one study carried out a complete analysis of the microbiome (bacteria, fungi, viruses, and parasites) (35). The two neonatal studies focus on bacterial identification (36, 37), while the pediatric studies focus on the identification of the virome (*n* = 1) (34), total microbiome (bacteria, viruses, fungi, and parasites) [*n* = 1] (35), and bacteria (*n* = 1) (33).

Studies looking at pediatric sepsis included patients immunocompromised from cancer treatment with sepsis in the pediatric ICU (*n* = 1) (33), community-acquired sepsis evaluation with negative cultures (*n* = 1) (34), and patients admitted to the PICU (Pediatric Intensive Care Unit) for sepsis and negative cultures (*n* = 1) (35).

Interestingly, in the three retrospective cohort studies and the two case report studies, mNGS analyses were performed retrospectively, but with the aim of evaluating the ability of mNGS to detect the etiologic cause of sepsis where previously the culture result was negative. Additionally, in the Anh study (34), the objective was to evaluate the viral etiological causes of sepsis in pediatric patients with SIRS due to sepsis and negative cultures. All studies with metagenomic technology identified microorganisms where cultures had been defined as negative or the cause could not be identified. Therefore, the results of the included studies warn that the identification of the microorganisms that cause sepsis in these populations can be improved with the use of the mNGS methodology. In addition, two studies allow us to think of viral, fungal, and parasitic aetiologies as the cause of sepsis. Additionally, most studies describe a high prevalence of polymicrobial infection in children with sepsis (33, 35).

About the sequencing platform used, sequencing was performed using the Illumina in four studies: HiSeqX (36), MiSeq (37), HiSeq2500 (33), MiSeq (34) and in one study, the Yan et al. (35), sequencing was performed using BGISEQ-50.

### Assessment of quality and risk of bias of the studies

In general, the risk of bias for observational studies was moderate to high: on average, 51% of the items did not meet the required information (Table 3A). The main problems that were common in the studies and that increased the risk of bias are with the methods and designs of the study, in the setting, bias, sample size, and statistical analyses. For example, the non-use of external negative controls (increasing the risk of reporting contaminating or non-disease-causing microorganisms), especially in the Anh study (34), which used non-sterile samples (swab and feces); a weak or absent explanation about the criteria used to describe the role of the isolates in the disease (pathogenicity of the isolates); the time between sample storage and analysis (criterion that can trigger changes in the relative representation of bacterial taxa and variability in metagenomic data) was not reported; samples were analyzed retrospectively, since the metagenomic analysis for identification was not planned from the beginning of the study and 100% of the studies involved retrospective analysis of the samples collected in the framework of studies originally designed for other outcomes; none of the studies calculated the power of the sample, and in the statistical analyzes, sensitivity and specificity analyses and the clarification or resolution of the species or strain were not performed.

**Table 3A:** Risk of bias reporting of observational studies: STROBE-metagenomics*

Item	Author		
	Horiba et al.	Anh et al.	Yan et al.
**Titles and abstracts**			
The term metagenomics should be included in the title or abstract, and the keywords of the study when these methods contribute to the results reported	●	●	●
**Describing methods and study design**			
Describe specimen collection, handling and storage processes, and nucleic acid extraction methods	●	●	●
Describe sequencing methods, including sequencing depth	●	●	●
Describe methods used for bioinformatics analysis	●	●	●
Describe quality assurance methods, including internal and external quality controls	●	●	●
Describe the use of orthogonal methods to confirm pathogen identity, function, and viability	●	●	●
Describe the criteria used to assess the role of pathogens in disease etiology	●	●	●
State the time from collection to results and cost consideration	●	●	●
**Setting**			
State whether sample collection was retrospective or prospective	●	●	●
**Participants**			
Consider factors influencing microbiota compositions when selecting participants	●	●	●
**Bias**			
Address potential sources of bias (sampling, transport, storage, library preparation, and sequencing)	●	●	●
Address potential bias introduced by bioinformatics analysis	●	●	●
Describe or address limitations of reference databases	●	●	●
**Study size**			
Describe clearly how power calculations were made	●	●	●
**Statistical methods**	●	●	●
State the limit of detection, including analytical sensitivity and specificity	●	●	●
**Discussion**			
Attempt or acknowledge the need for functional or phenotypic validation	N/A	N/A	N/A
Consider the need for species or strain resolution	●	●	●
**Other information**			
Report any ethical considerations with specific implications for metagenomics	●	●	●

* STROBE extension statement to guide the reporting of metagenomics studies ● Yes ● No

Regarding the setting of the studies, in three, an initial prospective design was proposed for the inclusion of the patients and the collection of the sample for the primary objective of the study, however, in all the studies, the mNGS analysis of the collected samples was conducted retrospectively, on samples stored and frozen. Similar situations in the two case studies. Additionally, the studies did not report the sample storage and transportation procedure from collection to processing. These situations are important given the risk of bias and quality of the studies. The time from sample collection to processing, including cold-chain transportation and transit, can affect the compositional profile of microorganisms inferred from metagenomics (overgrowth or degradation) (26). On the other hand, the recommendations promote reporting on the sequence of procedures in relation to whether the collection and analysis of the sample was prospective or retrospective. This would have an implicit risk of bias in the results. The analyte can degrade if there is a long time in between sample collection and the metagenomics assay (26).

For case studies, the quality of the studies was better (Table 3B).The main problems were found in the description of the cases, which lacked the clarification of clinical data, concomitant diseases, etc., and in the specification of the sampling concerning the moment of the analysis.

**Table 3B:** Risk of bias report for CARE case report^a^

**Title**	**Keywords**	**Summary**				**Introduction**				**Patient information**	**Clinical findings**	**Calendar**	**Diagnostic evaluation**				**Therapeutic intervention**	**Follow-up and results**				**Discussion**				**Patient's perspective**	**Informed consent**
		Introduction	Main symptoms and clinical findings	Diagnostics, therapeutic interventions, and results	Conclusion	Demographic information	Main symptoms	Medical, family, and psychosocial history	Concomitant diseases				Diagnostic methods	Diagnostic problems	Diagnostic rationale	Prognostic characteristics		Results	Follow-up test results	Observations	Adverse and unforeseen events	Strengths and limitations	Discussion of t medical literature	Justification of the conclusions	Main lessons		
Horiba	●	●	●	●	●	●	●	●	●	●	●	●	●	●	●	●	●	●	●	●	●	●	●	●	●	●	●
Mariya	●	●	●	●	●	●	●	●	●	●	●	●	●	●	●	●	●	●	●	●	●	●	●	●	●	●	●
Complete information					●						Incomplete information				●						Not reported				●		

^a^ CARE: CARE Guidelines for Case Reports.

### Characterization of the etiology in the neonatal population

Two studies address the characterization and impact of mNGS in the identification of microorganisms in neonatal sepsis. One is a report of two cases in neonates, and the other is the study and bacterial identification of placentas in premature neonates attributed to infection. On the other hand, although the characterization by gestational age (term and preterm) and the type of sepsis (early and late) was planned, there was a limitation in the reporting of these topics, given the absence of studies that evaluate this topic. Therefore, with the current information of the included studies, it is not possible to accurately describe the etiological characterization of neonatal sepsis by methods based on mNGS technology.

Horiba et al. (36) report the case of two neonates with sepsis and meningitis due to group B Beta hemolytic *Streptococcus* (GBS). Both neonates had no risk factors for sepsis and negative rectovaginal culture in the mother. They attended at 22 and 55 days of life due to clinical sepsis. In the diagnostic evaluation, the study of cerebrospinal fluid (CSF) suggested neuroinfection, but blood and CSF cultures were negative. Likewise, the PCR for GBS was negative in one of the cases. Therefore, both neonates received empirical treatment for three weeks. In addition, in the second neonate, E*nterococcus faecalis* was isolated in urine culture, resulting in targeted treatment and discharge, with torpid evolution and readmission after a week. Again, blood and CSF cultures were negative. In both cases, the identification of GBS was obtained retrospectively through metagenomic studies. Specifically for case two, the identification was in the sample of the first admission. It is important to note that given the negativity of the traditional studies performed (culture), prolonged exposure to antibiotics was performed, and treatment was not directed against the etiological pathogen. Likewise, in the identification of microorganisms by mNGS in the analyzed samples, approximately 16 microorganisms were obtained with different relative abundances (Table 1), both in the CSF and serum samples. For example, in case 1, the microorganisms with 90% relative abundance were *treptococcus*, and in case two, the microorganisms identified with the highest relative abundance were *Corynebacterium*, among others.

On the other hand, the study by Mariya et al. (37) analyzed placental samples from three cases of chorioamnionitis and preterm birth and one from a full-term birth without pathology as a negative control for infection. Taxonomic analysis showed that *S. pyogenes* was the most prevalent bacterium (69.1%) in the placenta of case 1, and the second most common species was *Burkholderia* spp (18.2%). However, *Burkholderia* spp. was also detected in the normal placenta (68.0%), suggesting contamination. On the other hand, in the other two cases of chorioamnionitis in advanced stages and needing urgent caesarean sections far from term, they identified a wide variety of species suspected of dysbiosis, but no specific bacteria causing the maternal or neonatal infection could be suggested (Table 1). The authors suggest the need to expand this knowledge and the study of maternal and/or neonatal complications due to infections of unknown origin.

### Characterization of the etiology in the pediatric population

The included studies use clear criteria for defining sepsis, involving the inflammatory response within its characterization. The ability of mNGS to improve microbial identification in previously negative culture samples and to correlate in more than 95% of cases with culture isolates is observed in all three studies. In fact, the isolates using mNGS identified microorganisms not commonly considered or defined as the cause of the systemic inflammatory response and infection. Horiba et al. (33) establish the objective of identifying the etiological diagnosis of sepsis due to mNGS in a group of pediatric patients immunosuppressed due to cancer treatment with intravascular catheter and bacteremia admitted to the pediatric ICU in a prospective cohort study. It includes two analysis groups, one with pathogen detected by culture (*n* = 12) and another group with sepsis and negative culture (*n* = 23). The NGS methodology detected all the bacteria at the family level, identified in culture. However, the dominant bacteria in mNGS were identical in only 8 of 12 patient cultures, raising the possibility of blood culture contamination with possible false positives.

On the other hand, in this study, several index values (in the mNGS analyses) were evaluated for diagnostic purposes: bacterial reads per million reads of the sequencing depth (BR) and relative importance values of the dominant bacteria (P1) were calculated to determine the clinical importance in etiological diagnosis causative bacteria, comparing mNGS analyzes with the results of blood cultures in patients with bacteremia. They propose BR index greater than 200 as an important index in the identification of the causal agent, showing an increase in BR in the presence of a greater number of bacterial genomes. In addition, the BR is greater at the beginning of bacteremia than after the start of treatment and follow-up. They also use a P1 value greater than 0.5 to determine the pathogens causing the infection, establishing that in patients with bacteremia bacterial, readings are significant and can be a useful technology in the diagnosis and management of the patient, contributing not only to the etiological identification but also to the follow-up and quantification of the response to treatment, also allowing the evaluation of the prognosis and effectiveness of the treatment through the follow-up of the abundance indices. The study by Anh et al.(34) aims to characterize, through mNGS, viral causes as an etiology in community-acquired sepsis with negative cultures. It reports that in 54% of the patients (*n* = 402/749) no isolation was obtained by culture. Of the 402 samples without isolation, 386 were evaluated by mNGS. Interestingly, viral sequences were identified in 93% of the samples from these patients, and in 13 patients, the identification of more than one virus was reported. The identified viruses were categorized according to whether they were known to cause disease and their prior identification (known to cause human infections, unknown pathogenicity, and previously unreported viruses).

The most frequently isolated viruses with known pathogenicity were enteroviruses, Hepatitis B virus, and Cytomegalovirus (CMV) [Table 1]. Previously reported viruses were detected in 3.4% of cases but with no known pathogenicity in humans. In children, the most frequent isolation was respiratory infection (RSV and HRV), followed by infection of the central nervous system (enterovirus). However, as a limitation of the study, a causal association between sepsis and the identified viruses cannot be established. More specifically, on several occasions, viral detection in non-sterile materials, such as respiratory samples and stool samples, may simply reflect the transportation of these viruses in those body compartments rather than a causal clinical association. Even so, the authors state that the results obtained allow the argument of viral etiology as a cause of community-acquired sepsis with negative cultures.

In the study by Yan et al. (35), retrospective analyses of mNGS for the identification of the complete microbiome (bacteria, fungi, viruses, and parasites) were carried out in serum samples collected and frozen in children with sepsis criteria admitted to the pediatric intensive care unit. A total of *n* = 34 children were included, of whom *n* = 18 had culture-confirmed sepsis and are categorized into two phenotypes for molecular and clinical analyses: community-acquired pneumonia and hospital-acquired pneumonia.

Indices of microbial ecological diversity, including species abundance, species count, Chao1 diversity index, and Shannon diversity index, were also performed in this study. Microbial ecological diversity was compared between the two phenotypes of children included, observing a greater number of bacterial species in hospital-acquired pneumonia. Interestingly, the significant correlation between the value of procalcitonin (understood as a biomarker for diagnosing bacterial sepsis) and the abundance of bacteria is described.

Of all the microorganisms identified, the potentially pathogenic ones were the least frequent and included *Escherichia coli*, *Pseudomonas aeruginosa, Acinetobacter baumannii*, and *Klebsiella pneumoniae*. Correlation analyses of abundance (the value of readings per million—RPM) of candidate pathogenic bacteria and important clinical phenotypes observed a correlation between the candidate pathogenic bacteria with the presence of septic shock and an association between the range of value of the bacteria richness (Chao1) and children with hospital-acquired pneumonia, with a significantly higher Chao1 value in bacteria with this phenotype compared to those with CAP.

The study also reported the identification of pathogenic viruses in the blood of children with sepsis in the PICU (human mastadenovirus B, cytomegalovirus, and Epstein-Barr virus) in only 14% of the children. Also interestingly, virus identification by mNGS was significantly and positively correlated with indicators of serum inflammation [C-reactive protein and interleukin (IL) 6], and the relative abundance of CMV was positively correlated with the presence of septic shock.

Concerning fungal identification, 19 potential pathogenic fungi were reported and were present in 35% of the patients analyzed. *P. jirovecii* was significantly abundant and negatively correlated with white blood cell count.

The study by Anh (34) merits special mention, in relation to the analysis of non-sterile samples for the etiological identification of viruses in patients with sepsis. The study of these samples has a challenge and a limitation in being able to assume that the identification of the virus may be the etiological cause of sepsis and distinguishing pathogens from commensals or contaminants, therefore, caution must be taken to not impute the etiological diagnosis of sepsis to the viruses identified in these samples. Confirming the presence of microbial DNA or RNA in association with disease is an important step in establishing a causal relationship between a microorganism and disease in this type of mNGS study (26). Faced with this challenge, the authors carry out a description and analysis of the viral sequences detected in non-sterile samples based on sequences of known pathogenic viruses, viral sequences with no known pathogenicity, sequences related to contaminants, and/or not previously reported in humans. However, it should be noted that there is no description of the criteria used in the metagenomic analyses to establish the causality of these sequences with the disease; this is a very important limitation in the objective of characterizing the viral etiology in sepsis with negative cultures.

## Discussion

The systematic review addresses the importance of mNGS in the identification and characterization of sepsis-causing germs in neonatal and pediatric ages and it is the first that is carried out with this objective in this age group. In all included studies, it was possible to identify microorganisms in a high proportion of cases of patients with sepsis and negative cultures, regardless of the immune status or severity of the disease. It is also relevant to observe that in this scenario of sepsis with negative cultures, the mNGS can detect microorganisms other than bacteria, such as viruses and fungi, in a single sample. The scant research into the etiology of neonatal sepsis using mNGS technologies also exposes the need for research in this field, as does the demand to improve the quality of research and reduce bias by adhering to guidelines such as STROBE-metagenomics.

Similarly, the data from the systematic review demonstrate that through the mNGS it is possible to analyse and study several indices that help monitor the response to treatment, the diagnosis, and the identification of the etiological cause of sepsis. This information is useful given the use of these indices in clinical practice it serves to propose the development of integral diagnostic methods in sepsis and in general in infectious diseases (33, 38). Also, a correlation is observed between the indices of ecological diversity (abundance) with serum biomarkers previously proposed in the literature for the diagnosis of bacterial and viral sepsis; a positive correlation with the Chao1 index in parasitemia was even reported in patients with known immunodeficiency. On the other hand, the mNGS allows the study of resistance genes in the identified microorganisms, which further guides antibiotic treatment (27, 39, 40). This points to the additional importance of mNGS in the management of patients with sepsis in the neonatal unit and pediatric ICU, allowing the orientation and timely adjustment of antibiotic treatment.

Using mNGS allows the identification of the viruses, bacteria, fungi, or protozoa causing sepsis in the processing of a single sample and in an unbiased way. This is of vital importance in the therapeutic orientation and diagnostic approach to critically ill children. One of the included studies (33) identified *P. jirovecci* in the blood of patients in pediatric intensive care. This germ can cause severe lung disease that leads to hypoxemic respiratory failure and acute respiratory distress syndrome (ARDS) in children with immunodeficiency. In fact, in this study, the abundance of *P. jirovecci* in the blood of immunocompromised children was significantly higher than in immunocompetent children in critical care. These types of findings correlated significantly and negatively with the white blood cell count. The importance of these types of findings in pediatric sepsis is transcendental. With traditional methods, this type of isolation is more complex, which leads to the excessive use of broad-spectrum antibiotics that favour antimicrobial resistance and select the microbiota of these immunosuppressed (13, 41).

Similarly, mNGS would have the potential to identify the presence of viral or bacterial co-infection. A recent study found that viruses such as Rhino/Enterovirus have a high prevalence of coexisting with other viruses and patients–especially premature children with bronchopulmonary dysplasia and heart disease–with these simultaneous viruses are associated with worse clinical outcomes (42). Using mNGS could guide against the coexistence of these coinfections, which in risk groups is associated with worse outcomes. This is particularly important in the winter months or the rainy season because the simultaneous presence of viruses or bacteria is not easy to establish with cultures or traditional diagnostic techniques and would allow therapy to be targeted for these.

The information and data from studies in adults elucidate the importance of mNGS in certain scenarios. For example, studies in critically ill adults with sepsis have shown the advantage of mNGS in detecting etiologic organisms of sepsis where cultures were negative. In the study by Gu et al. (43) it was observed that this technology allowed the identification of microorganisms in the preponderance of negative cultures, identifying two additional pathogens to those detected by sterile site cultures and in all body fluid samples from patients with suspected sepsis and that previously the result culture had been negative. Similarly, in Zhang's study (44) in adult patients in bronchoalveolar lavage samples with community-acquired pneumonia, nNGS was superior to cultures in detecting pathogens in these samples. In addition, also in adults, the superiority of mNGS is observed compared to culture in the identification of microorganisms even in the presence of previous use of antibiotics (45). Additionally, Hu (46), revealed how a significant number of patients with negative cultures received antibiotics due to suspicion of bacterial sepsis; however, in the mNGS analysis it was possible to conclude that in these cases the etiology was viral, a situation that shows the benefit of this technology for guiding better antibiotic therapy. The data from this systematic review also shows the importance of mNGS in detecting microorganisms that are difficult to grow in traditional cultures and other than bacteria in neonatal and pediatric patients with clinical criteria for sepsis and negative cultures.

Regarding the time to obtain the result and adjustment of therapy from the clinical sample by mNGS, they also show promising results. For example, in the adult studies of Miao, Zhang, Gu, and Hu (43–46) it is observed how the time range from sequencing to identification is a few hours. Furthermore, Gen (47) and Guo (48) show in their results in adult patients with sepsis and in the intensive care unit the ability to adjust earlier antibiotic treatment directed at microorganisms with mNGS. Similarly, Geng (47) shows the high possibility of polymicrobial infection and Hu (46) timely suspension of antibiotics against viral etiologies in sepsis with negative cultures.

Nevertheless, although some studies in adults have shown that etiological identification and diagnosis by mNGS may have better sensitivity and specificity compared to cultures and traditional diagnostic methods, especially in the scenario with negative cultures (49, 50); it is also true that other studies in adults show that the diagnostic performance may not be superior to traditional diagnostic methods against common bacterial etiologies, especially those grown in usual cultures used in the clinical laboratory (45, 51). However, the potential of the technology lies in the identification of microorganisms that are difficult to grow and viral, fungal, and parasitic etiologies in a single clinical sample and in a single procedure time (27, 54, 55). In addition, with the improvement of technology in shorter times.

The data from this systematic review of the literature, as well as that proposed by other authors, allow us to infer and propose mNGS as a promising tool for the detection of pathogens in clinical samples from patients with sepsis improving clinical diagnosis (51). However, although the molecular methods used in the diagnosis in microbiology have allowed improving the times of the results in comparison with the cultures (54), it should be kept in mind that currently the tests based on mNGS are expensive and are not standardized in clinical laboratories for care at the patient's bedside (51), a situation that leads to increased costs and time to obtain results. Therefore, studies are required that allow more rapid implementation of the technology in the clinical care of the patient and shorten the turnaround time for the library preparation and the runs on the NGS platforms (54). It is expected that in the coming years, there will be a massive implementation of mNGS technology, automating and standardizing laboratory processes that allow results with shorter response times (51).

In summary, the recent guidelines for the management of sepsis in children and newborn recommend starting antibiotics in the first three hours after sepsis is diagnosed and in the first hour after septic shock is identified (15, 54, 55). However, although this intervention is part of the initial measures package known as the Bundle, in all cases it is started empirically, and broad-spectrum antibiotics are usually used to cover the possible etiological agents. With traditional methods, it is not common to be able to identify the causal germ (or it is often viral), which leads to long courses of antibiotics being administered unnecessarily. The routine use of mNGS would allow a more rational use of antibiotics, administering shorter and targeted cycles with all the beneficial effects in terms of resistance and microbiota selection that this would have.

Another aspect that we consider important is that mNGS has been validated for the detection of pathogens in normally sterile fluids such as cerebrospinal fluid, blood, and even amniotic fluid, as described in one of the investigations included in this systematic review (35). This would make it possible to identify germs that are usually elusive or that traditional methods do not detect due to their low bacterial count or because they are difficult to access, such as abscesses or microbial collections in deep anatomical areas, etc.

We consider it important to keep in mind that this technology probably does not replace culture 100% in the diagnosis and clinical management of the patient, but rather complements it, especially in the initial empirical orientation of the treatment and its adjustment in the course of the disease.

The limitation of the number of studies that address neonatal sepsis (together with the premise that neonatal sepsis is one of the most frequent pathologies in the neonatal unit), the high burden of sepsis “with negative cultures,” the antibiotic overexposure in this group, and the clinical consequences of the use of antibiotics in neonates makes it interesting and necessary to address this research problem by using mNGS technologies in the identification and diagnosis of neonatal sepsis. This would allow evolution towards the clarification of the etiology and true prevalence of sepsis, even clarifying the participation of commonly used microorganisms that are difficult to grow in the laboratory, thus reducing the adverse consequences of the use of antibiotics in the neonatal population, especially extreme preterm births.

The initial objective of the systematic review was aimed at the etiological characterization of sepsis in neonates and pediatric patients by mNGS. However, we note that, in the current state of knowledge, the studies carried out with this technology are mainly focused on the ability to detect microorganisms in patients with sepsis and negative cultures. This may be because researchers are interested in evaluating the ability of mNGS to improve etiological diagnosis in this type of patient. Likewise, it was also observed that due to the methodological quality of the studies there is an implicit high risk of bias in the results. These issues open an interesting field of research and in the methodological way of approaching future studies. It is recommended that future studies adhere to methodological quality recommendations, such as the STROBE-metagenomics instrument. In addition, it is highly recommended that mNGS analyses be the primary objective in initial study planning, with the aim of a comprehensive and unbiased understanding of the microbial etiology of sepsis in these age groups. This could impact knowledge in this field and reorient management guidelines in the initial empirical choice of antimicrobial treatment.

In the same way, recognizing the capacity of mNGS technologies to improve diagnosis through the detection of pathogens and the discovery of new microorganisms and the capacity to improve the treatment and follow-up of the patient with the identification of resistance genes and the discovery of new biomarkers, the broad field of research with these technologies in neonatal and pediatric patients is interesting. In this sense, it was observed that only one study (33) evaluated resistance genes and only two reported in the mNGS analysis several index values (BR and P1) and ecological diversity indices (33) that proved to be useful in the diagnosis, identification of the causative pathogen of sepsis and monitoring of treatment response in patients.

Another advantage of the bedside clinical care approach with mNGS is that it also allows non-selective amplification of targets, obtaining unbiased information on microorganisms, and identification of resistance markers, virulence factors, and host factors in one sequence run (27, 29, 39). Taken together, these advantages of mNGS would allow optimizing the clinical management of critically ill and high-risk patients in the future, such as in the neonatal and pediatric ICU, resulting in a decrease in morbidity and mortality. Therefore, work aimed at developing strategies that allow the implementation of this technology at the patient's bedside is essential, allowing personalized and translational medicine in pediatric and neonatal sepsis from the systems biology approach.

We consider that our study has several limitations. The small number of studies, the low quality of the evidence, and the diagnostic and methodological heterogeneity precluded a quantitative meta-analysis. However, we consider that a qualitative approach gives us a general overview of the usefulness of mNGS in the setting of neonates and children with sepsis, particularly identifying the impact on the rationalization of antibiotic therapy in this group of patients and the clinical benefits derived from a rational use of antibiotics in critically ill neonates and children. Another limitation is that with this technique, it is difficult to distinguish between pathogens and commensals typical of each anatomical site. The challenge is for the clinician to correctly interpret each isolation according to the previous immunological status of each patient and their comorbidities. We believe that mNGS has great applicability and clinical utility in critically ill children with sepsis. More studies are needed to correlate the isolated germs with those described in the literature to date to deepen our knowledge of the current aetiological agents in the post-vaccination era, and thus conduct a more rational therapeutic orientation and avoid the clinical complications that the indiscriminate use of antibiotics use entails.

## Conclusions

The use of mNGS allows etiological identification in neonatal and pediatric sepsis to be improved, especially in the context of negative cultures and in the identification of unusual microorganisms (bacteria that are difficult to grow in culture, viruses, fungi, and parasites). The number of investigations is currently limited, and the studies are at high risk of bias. Further research using this technology would have the potential to improve the appropriate use of antibiotics.

## Data Availability

The original contributions presented in the study are included in the article, further inquiries can be directed to the corresponding author/s.
